# The Use of Noncommercial Parent-Focused mHealth Interventions for Behavioral Problems in Youth: Systematic Review

**DOI:** 10.2196/51273

**Published:** 2024-09-24

**Authors:** Katherine I Magnuson, Kexin Li, Grace Beuley, Stacy R Ryan-Pettes

**Affiliations:** 1 Department of Psychology and Neuroscience Baylor University Waco, TX United States

**Keywords:** behavioral parent training, mobile health, mHealth, mobile app, adolescent, substance use, child mental health condition, mobile phone

## Abstract

**Background:**

The rates of substance use among adolescents are alarmingly high, and current treatment options lack integration of parent-focused interventions, despite evidence that effective parenting practices can mediate treatment outcomes for adolescents involved in substance use. Accessibility and other barriers to parental interventions may be mitigated through mobile health (mHealth); however, few mHealth platforms target substance use behaviors for adolescents through the implementation of behavioral parent training strategies.

**Objective:**

This study seeks to review current mHealth platforms within empirical literature that are designed to increase effective parenting through behavioral parent training techniques. Because of the paucity of mHealth modalities that use parenting strategies to target substance use in adolescents, the objective was expanded to include mHealth platforms addressing behavior problems among youth, given that parent-targeted treatments for these clinical presentations overlap with those for adolescent substance use. Overall, the systematic review was conducted to inform the development of mHealth apps for parents of youth involved in substance use, improve accessibility, and better align with parental needs.

**Methods:**

This systematic review was conducted using the PRISMA (Preferred Reporting Items for Systematic Reviews and Meta-Analyses) method to select relevant articles across several databases. Each study was assessed for relevance and inclusion. Each study was reviewed for demographics, delivery medium, intervention status as stand-alone treatment or as an enhancement to treatment, mobile device used, mental health condition targeted, intervention type, underlying intervention theory, behavior change theory applied in design, behavior change techniques, parent training techniques, youth outcomes, parent outcomes, visual design, content, and features.

**Results:**

Overall, 11 studies were included. Nearly all studies (9/11, 82%) predominantly sampled female caregivers. Most of the studies (6/11, 55%) integrated social learning theory. Only a few of the studies (2/11, 18%) discussed the embedded behavior change theories, whereas all the studies (11/11, 100%) used at least one behavior change technique to encourage change in parental behaviors. Many of the studies (7/11, 64%) tailored design features to the end user. Of the various behavioral parent training techniques, nearly all studies (10/11, 91%) included the skill of strengthening the parent-child relationship. A preliminary evaluation of treatment outcomes suggests a positive impact of parent-targeted mHealth interventions. When reported, the effect sizes for treatment ranged from Cohen *d*=0.38 to Cohen *d*=1.58 for youth and from Cohen *d*=0.13 to Cohen *d*=2.59 for parents.

**Conclusions:**

Although features and techniques were referenced, only a few of the studies provided specific information related to behavior change theory (2/11, 18%), visual design (2/11, 18%), and the translation of parent-targeted interventions to mHealth platforms. Such information would be useful for the development of mHealth apps. Preliminary outcomes for youth and parents are encouraging, but future studies should consider conducting a meta-analysis as the body of studies grows to determine aggregate statistical findings.

## Introduction

### Background

Adolescent substance use occurs at alarming rates in the United States, with approximately 4.3 million youths using illicit substances in 2019 [1]. Despite evidence indicating that 1.1 million of these youths needed substance use treatment, <1% obtained treatment [1]. For the few youth who receive substance use treatment, parent-focused interventions, shown to improve parenting practices that mediate adolescent outcomes, are often a missing component [2-4]. This is concerning because there are limited resources and pathways of access for parents of adolescents involved in substance use to receive parenting resources or support [5-7].

This inability to access parent-focused interventions may be related to both a lack of availability of these interventions [8,9] and logistical, personal, or systemic barriers to treatment engagement [10-15]. Nonetheless, less frequently acknowledged is that the currently available treatment options for parents of adolescents involved in substance use may not embody the type of treatment that these parents desire. Recent research showed that, among parents of youth in treatment for substance use, the majority (72%) perceived a need for parent-focused services related to parenting their adolescent child after substance use treatment; when aftercare was offered via mobile phone, this figure increased to 91% [16]. One interpretation of these findings is that parents are not currently receiving support through their preferred medium.

Taken together, these findings highlight the need for greater access to strategies for engaging in effective parenting of children with a history of substance use, and leveraging mobile health (mHealth) may help address this service gap. Unfortunately, while the development of mHealth apps is moving at a rapid pace in most fields of health care, it lags in the area of adolescent substance use [17]. There is only 1 published study of an mHealth app for parents of youth involved with substances [18]. However, this app focuses on delivering mindfulness interventions and excludes a focus on behavioral parent training. Given the demonstrated benefits of behavioral parenting approaches in curtailing adolescent substance use [4,19] and the potential advantages that mHealth apps offer in broadening access and reach, it is surprising that more attention has not been paid to developing an mHealth intervention specifically for parents of youth involved in substance use.

This systematic review seeks to evaluate mHealth apps in empirical literature designed to increase effective parenting through behavioral parent training techniques for behavior problems in their child. Given the overlap in behavioral parent training interventions for behavior problems and substance use [20-23], the results of this review could inform the development of future parent-focused mHealth apps for parents of youth involved in substance use, improving accessibility and matching parental desires for treatment mediums [24,25].

### Behavioral Parenting Practices and Adolescent Substance Use

Parenting practices shape the development and outcome of a child [26]. The literature is replete with results showing that ineffective parenting practices such as poor monitoring and supervision, inconsistent discipline, poor limit setting, and low positive parenting are associated with a range of behavior problems [27], including substance use disorders [28,29]. On the basis of the plethora of research demonstrating the importance of effective parenting practices, evidence-based treatments designed to treat behavior problems among adolescents, including substance use, are heavily steeped in addressing ineffective parenting using behavioral parent training [20,22]. Broadly, behavioral parent training is an evidence-based approach to helping parents apply behavioral strategies to improve their child’s behavior and increase positive family interactions; it is also referred to as parent management training and parenting training [30].

### mHealth Apps for Behavioral Parent Training

After conducting a literature review, Jones et al [31] concluded that behavioral parent training is a strong fit for transfer to technological mediums such as smartphone apps. We concur and argue that behavioral parent training is compatible for translation to mHealth because key parenting strategies in the behavioral parent training protocols can be aided with smartphone apps that include design features tapping into general principles of behavior change to promote parenting behaviors. Specifically, app features such as routine prompts and timely notifications with tips may promote consistent implementation of rules, facilitate limit setting, and support the use of consistent discipline. In fact, prompting through push notifications aligns with behavior change theories in mHealth that emphasize the use of reminders to enact skills and the integration of motivational support [32,33] to foster the consistent use of learned, effective parenting practices; for example, the use of encouragement may include periodic messages that remind parents of a learned parenting skill and encourage them to continue using the skill.

### Objectives

The original aim of this study was to systematically review available noncommercial mHealth apps for parents of youth involved in substance use. However, the limited literature on mHealth apps providing parental intervention to target adolescent substance use made this aim challenging. In an effort to continue to explore and review this subject despite the scarcity of research, the objective of this study expanded. In particular, the focus shifted slightly to a systematic review of mHealth apps that provide behavioral parent training or components of behavioral parent training to enhance the use of effective parenting for behavior problems in youth. This shift broadened the search for current mHealth apps in the literature, while also maintaining relevance and applicability to parent-targeted mHealth interventions for adolescent substance use. More specifically, the behavioral parenting interventions that have been implemented to target youth behavior problems significantly overlap with those used to intervene on adolescent substance use. The large overlap in treatment content may be related to the notion that adolescent substance use is often conceptualized through a broader lens of behavioral problems in youth [20-23]. Therefore, the results of this review could generalize to the development of future parent-focused mHealth apps for parents of youth involved in substance use [24,25].

This study sought to answer four main research questions:

1. What are the general characteristics of behavioral parent training apps under development?
2. What is the empirical evidence underlying behavioral parent training apps under development?
3. What are the main parenting strategies covered in behavioral parent training apps under development?
4. What implications do the characteristics, empirical evidence, and parenting strategies evidenced in current behavioral parent training apps have on the development of an mHealth app for parents of youth with behavior problems involved in substance use?

To answer these questions, we summarized the major design elements, features, content, and theoretical foundations of the evaluated apps and paralleled these with the components of substance use treatment to provide recommendations for the design of mHealth apps for parents of adolescents who use substances. In contrast to existing studies, these objectives enhance knowledge about apps tailored specifically for parents of adolescents who use substances.

While prior studies have reviewed mHealth apps based on behavior change theory and techniques, they included a narrow focus on these factors [33-35] and did not review behavioral parenting practices. Some studies have reviewed commercial parenting apps [17,36], apps for specific groups of parents (eg, fathers with low-income status and new parents [36-38]), or apps for parents with adult children [39]. However, these studies did not review apps designed to teach behavioral parenting skills to address behavior problems exhibited by their child. Indeed, several reviews provide information about the effectiveness of technology-based interventions for behavior change and for parents of children with emotional or behavioral issues [40-42]. However, these reviews included a mix of dated mediums alongside mHealth apps, including websites, software, videoconferencing services, and SMS text messaging.

## Methods

### Literature Search

The search was conducted electronically in English between June and September 2019, again in March 2021, and once more in October 2022. No restrictions on the date or year of article publication were imposed in the original 2019 search, and the 2021 and 2022 searches were limited to materials published in the time since the prior searches. The following databases were used: PsycINFO, MEDLINE (PubMed), Google Scholar, Scopus, Web of Science, and WorldCat. References from selected articles and past literature review articles were also examined to identify potential sources that may have met our criteria for this review [39,40,43,44].

The following mobile technology search terms were used: *mobile phone*, *mHealth*, *eHealth*, *SMS*, *text messaging*, *mobile application*, *tablet*, *smartphone*, and *cell phone*. The following parent treatment search terms were used: *parent training*, *intervention*, *treatment*, *parent management training*, *parent-child interaction*, and *behavioral training*. The following mental health search terms were used: *behavior*, *attention-deficit/hyperactivity disorder*, *autism spectrum disorder*, *posttraumatic stress disorder*, *trauma*, *psychological*, and *disorders*. These terms were entered into databases using various search combinations, including (mobile phone OR cell phone OR smartphone OR tablet) AND (parent train* OR treat* OR parent management train*) AND (behav* OR trauma OR disorder OR attent* OR psycholog* OR autism) AND (SMS* OR text messag* OR application OR mHealth OR eHealth).

The search conducted in October 2022 to update the results used the original search terms with date restricted to the years since the search conducted in March 2021 (ie, 2021-2022). In the search update, searches in 3 databases were modified to limit the number of results for relevance. Specifically, in MEDLINE (PubMed), the search was limited to clinical trials and randomized controlled trials; in Scopus, it was limited to articles; and in Web of Science, additional search criteria—adol* OR child* OR parent* OR caregiver OR mother OR father OR youth—were applied to filter out irrelevant results.

### Study Selection and Eligibility Criteria

Due to the paucity of studies in this field, the titles and abstracts identified from the search process included both peer-reviewed feasibility or acceptability articles and conference proceedings. Articles were screened against predefined inclusion criteria (Textbox 1) by 3 reviewers (SRR-P, KIM, and KL), who independently conducted the search and met afterward to integrate the search results and make joint decisions about inclusion and exclusion for each record.

**Criteria for eligibility.**

**Inclusion criteria**

- The study investigated parent-targeted interventions to influence child mental health conditions (defined as the presence of adverse behavioral and emotional symptoms that may be contributing to psychological difficulties). These conditions may include disruptive behaviors and conduct disorder symptoms, substance use, attention-deficit/hyperactivity disorder symptoms, trauma symptoms, and autism spectrum disorder symptoms; however, developmental, language, speech, and motor delays were excluded because these may not always be directly related to psychological symptoms.
- The study provided data on the efficacy or effectiveness of the intervention.
- The study provided data on either parent or child outcomes.
- The interventions only used mobile or tablet devices (studies were excluded if they involved the use of websites or computers in any capacity).
- The intervention content, such as specific parenting skills, was delivered via SMS text messaging or mobile apps (as opposed to professionals delivering interventions via mobile devices).
- Either stand-alone treatments or enhancements to existing treatments were included if the intervention involved more than simple reminders to attend regular treatment, based on the rationale that even enhanced treatment components may serve as stand-alone interventions with further research development.
- Studies involving biological parents, nonbiological parents, and foster caregivers were included.
- Studies that involved interventions targeting parents of children ranging in age from 2 to 18 years were included, based on the rationale that regardless of differences in implementations depending on the age of the child the basic principles of certain effective parenting practices (eg, parental monitoring) remain consistent.
- The articles or conference papers were in English.

### Identification and Description of Study Characteristics

#### Study Characteristics Assessed

Each article selected for the review was assessed for various characteristics, including demographics, delivery medium, intervention status as stand-alone treatment or as an enhancement to treatment, mobile device used, mental health condition targeted, intervention type, underlying intervention theory, behavior change theory applied in design, behavior change techniques, parent training techniques, youth outcomes, parent outcomes, visual design, content, and features. Each of these characteristics was operationalized according to this review’s context (Textbox 2).

**Operationalization of the characteristics and features reviewed.**

**Characteristics and operationalization**

- Delivery medium: the method used to deliver the intervention on the mobile device, which included the use of a mobile app, electronic monitoring wristbands, and the use of smartphone or tablet features such as SMS texting, video calls, and video recordings
- Stand-alone treatment: the intervention is administered solely via the mobile device without being administered alongside, or in conjunction with, in-person treatment
- Enhancement to treatment: the intervention is administered in person, and the mobile device is used as a supplemental feature of treatment
- Mobile device used: the type of mobile device used to deliver the intervention
- Mental health condition targeted: the adverse behavioral or emotional symptoms exhibited by the children of the population of parents studied
- Type of intervention used, incorporated, or adapted: the parent-targeted intervention used in the research study that can be fully used, shortened, selectively used, or adapted, with the primary skills being implemented
- Underlying intervention theory: the theoretical foundation of the parent-targeted intervention
- Behavior change theory applied in design: a method for understanding how variations in treatments or interventions can lead to changes in behavior (Hekler et al [45])
- Behavior change techniques: a range of 26 methods used in the design of the mobile intervention to change an individual’s behavior [46]; the definitions of these 26 techniques can be found in the taxonomy developed by Abraham and Michie [46]
- Youth outcomes: changes in youth symptoms or behaviors after parent-targeted intervention is administered
- Parent outcomes: changes in parent behaviors after parent-targeted intervention is administered
- Visual design: assessment of the visual quality and look and feel of the program, including aesthetics, layout, and size [43]
- Content: assessment of the material provided and learned in the program, including the use of evidence-based content, quality of information provision, completeness and conciseness, and clarity about the program’s purpose [43]
- Features: assessment of different aspects used in the design of the mobile intervention

These characteristics were first assessed through careful reading of each article by the lead author (KIM). If the relevant elements could not be identified through reading the article, the references of the article were reviewed to determine whether they were included in the preliminary work. The authors of 2 (18%) of the 11 studies were contacted to inquire whether further research surrounding the initial study had been conducted. One author responded to the inquiry. To identify behavior change techniques, visual design qualities, content, and theoretical foundations, the methodologies outlined in the following subsections were used.

#### Identification of Behavior Change Techniques

Interventions were evaluated for the types and number of behavior change techniques using the taxonomy of behavior change techniques developed by Abraham and Michie [46].

#### Assessment of Visual Design and Content

Visual design and content were evaluated using Enlight (MindTools.io), a 5-point rating system ranging from 1=*very poor* to 5=*very good*, developed for the assessment of eHealth interventions [43].

#### Identification and Assessment of Theoretical Foundations

Theoretical foundations of the treatments and mobile apps were assessed through the implementation of a theory coding scheme [47]. This coding scheme outlines various steps for classifying the presence of the use of theory in interventions [47]. In this review, theoretical foundations were coded as present based on their mention in the article or its references (ie, item 1 [47]). When referenced or mentioned by the study, the presence of a theoretical foundation was coded. When assessing behavior change theory in the design of the mobile interventions, it was noted when a theory was not specifically mentioned but only alluded to in the study. Specifically, when a study mentioned the use of theory without specifying the name of the theory or its characteristics, it was marked accordingly.

## Results

### Demographic Information and Designs of Reviewed Studies

**Figure 1:**
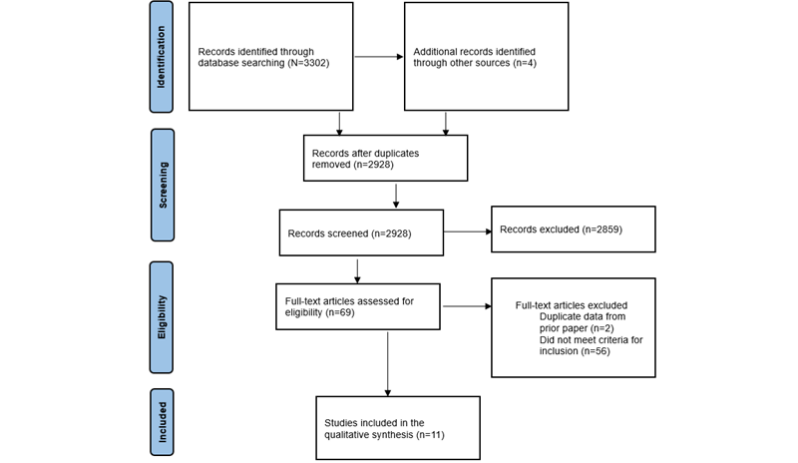
PRISMA (Preferred Reporting Items for Systematic Reviews and Meta-Analyses) flow diagram.

The PRISMA (Preferred Reporting Items for Systematic Reviews and Meta-Analyses) method was used to conduct the systematic search [48,49] (Figure 1), which included the PRISMA checklist that can be found in Multimedia Appendix 1 [50]. Through this process, a total of 11 studies were included in this review, and each was assessed for demographic information (Table 1). The earliest studies reviewed were published in 2014 [51,52]. Of the 11 studies, 7 (64%) were randomized controlled trials. The sample sizes ranged from 10 to 371 participants. Most of the parents included were mothers (ranging from 77% to 100%); however, in the study by May et al [50], the intervention was delivered to fathers. The parental ages ranged from 18 to ≥50 years. The target children’s ages ranged from 2 to 18 years. Each study recruited participants from a range of settings, including primary care clinics (1/11, 9%), community health agencies (4/11, 36%), social services (2/11, 18%), juvenile justice centers (1/11, 9%), early education agencies (1/11, 9%), community parenting support groups (2/11, 18%), autism organizations and intervention centers (1/11, 9%), child psychiatrist (1/11, 9%), schools (1/11, 9%), and social media platforms (3/11, 27%). Families were included if the child had “externalizing behavior problems,” “disruptive behaviors,” “symptoms of conduct disorder,” “autism spectrum disorder,” or “attention-deficit/hyperactivity disorder.”

**Table 1:** Demographic information of reviewed studies.

Study, year	Caregiver identification^a^ (%)	Child sex (%)	Parent age, y (%)	Child age (%)	Recruitment settings	Parental race or ethnicity (%)	Socioeconomic status (annual income; US $), (%)	Caregiver composition (%)	Treatment length
Breitenstein et al [53], 2016	Mother (94.9)	Female (57)	30-39 (63.3)	2-5 y (NR^b^)	Primary care clinic	African American (64.6)	>20,000 (65.8)	Not married (60.8)	12 wk
Feil et al [54], 2018	Female (77)	NR	Mean 44.7, SD 10.08 (NR)	8-12 y (NR)	Community parenting groups and social media	White (89)	>25,000 (29); 25,000-50,000 (32)	Two-adult household (63)	4 wk
Hemdi and Daley [55], 2016	Mother (100)	NR	Mean 32.9 (NR)	Mean 63.18 mo (NR)	Autism organizations and intervention centers	NR	NR	Married (90.62)	4 sessions
Jones et al [51], 2014	Female (71)	Male (57)	Mean 35 y (NR)	Mean 5.57 y (NR)	Schools and community health agencies	Ethnic minority (57)	NR (low income^c^; 100)	Single (57)	8-12 sessions
Lefever et al [56], 2017	Mother (100)	Male (56)	Mean 28.91 y (NR)	Mean 4.56 y (NR)	Social service agencies, early education agencies, and community health agencies	Hispanic (46); African American (33)	Mean 18,608, SD 15,835	NR	8 sessions
Mason et al [57], 2021	Female (90.4)	Female (67)	Mean 45.6 y (NR)	Mean 15.2 y (NR)	Community health agencies	White (84.6)	NR (low income^d^; 100)	NR	4 wk
May et al [50], 2021	Father (100)	NR	Mean 42 y (NR)	4-11 y (78)	Community parenting groups and social media	NR	NR (financial difficulty; 39)	Two-parent household (71)	16 wk
Pina et al [52], 2014	Mother (80)	NR	Mean 38.4 y (NR)	NR (K-12^e^)	NR	NR	NR	Two-adult household (100)	2 wk
Schaeffer et al [58], 2022	Female (100)	Male (55.9)	Mean 39.4 y (NR)	Mean 14.6 y (NR)	Social media and juvenile justice centers	White (76.5); Hispanic or Latinx (14.7)	10,001-20,000 (17.6); 20,001-30,000 (14.7); 50,001-60,000 (14.7); ≥60,000 (26.5)	Sole adult household (44.1); 2-parent household (29.4)	12 wk
Sonne et al [59], 2016	NR	Male (69.2)	NR	Mean 9.3 y (NR)	Community health agencies and child psychiatrist	NR	NR	NR	4 wk
Sullivan et al [60], 2019	Mother (95)	Male (55)	Mean 50 y (NR)	Mean 8.9 y (NR)	Social service agencies	White (95)	NR	Married (50); single (25)	10 wk

^a^Caregiver identification aligns with the report in the respective articles; identification as a mother or father should not assume gender.

^b^NR: not reported.

^c^Jones et al [51] define low income as the “adjusted gross income did not exceed 150% of the federal poverty limit, which takes into account both income and number of residents in the home.”

^d^Mason et al [57] did not provide specific financial ranges; however, the sample was described as “low income.”

^e^Pina et al [52] did not provide the ages of the children but specified that they were in grades K-12.

Of the 11 studies, 4 (36%) did not report the race or ethnicity of the sample. Among studies that reported race or ethnicity, the majority of the participants (ranging from 79% to 95%) were either from ethnic minority families (3/11, 27%) [51,53,56] or White (4/11, 36%) [54,57,58,60]. Information pertaining to socioeconomic status followed a similar pattern. Of the 11 studies, 5 (45%) included families who were identified as coming from a lower socioeconomic background [51,53,56-58], 2 (18%) included participants experiencing financial stress [50,54], and 4 (36%) either did not report income [52,55,59] or did not provide any socioeconomic information [60].

Of the 8 studies that reported the marital or partnership status of the parents, caregivers, or legal guardians, 5 (62%) reported that the majority (ranging from 50% to 90.6%) of the participants came from a 2-parent household (married couple or cohabiting couple).

Treatments were implemented for time periods ranging from 2 to approximately 16 weeks. Of the 11 interventions, 4 (36%) served as an enhancement to treatment, and 7 (64%) served as stand-alone treatments. Most of the studies (7/11, 64%) used a mobile app as a delivery medium.

### Theoretical Frameworks

Transferring treatment to a digital platform requires consideration of both the intervention’s theoretical framework and the theoretical frameworks that promote behavior change within a mobile platform.

#### Theoretical Framework of the Interventions

All studies (11/11, 100%) identified for this review drew from empirically based or evidence-based parent management training curricula, including the Chicago Parent Program, Behavioral Parent Training, Multisystemic Therapy, Behavioral Model Training, Helping the Noncompliant Child, and The Incredible Years Program. It is understood that the most prominent theoretical frameworks for these treatments include behaviorism (operant principles), the ecological systems framework developed by Bronfenbrenner [61], social learning theory, and the coercion model.

Of the 11 studies, 6 (55%) [51,53,56,57,59,60] explicitly discussed social learning theory, 2 (18%) [51,53] discussed the coercion model, 1 (9%) [58] discussed the social ecological framework developed by Bronfenbrenner [61], and 1 (9%) [55] discussed the transactional model of stress as the main theoretical framework for the parenting interventions included in the apps. Of the 11 studies, 4 (36%) did not expressly mention the coercion model as a theoretical framework, but this model was implied through information related to app content [52,57-59]; for example, these studies described implementing skills such as parental communication, effective parent-child interaction, monitoring, and limit setting to improve parental responses to child behaviors considered problematic, which is a tenet central to the coercion model [62]. Of the 11 studies, 2 (18%) did not expressly mention the guiding theoretical framework, and nor did they provide enough information to make inferences about the theoretical framework [50,54]. However, these studies did reference several behavioral parent training skills that are drawn from multiple interventions (eg, parent-child interaction therapy and parent management training).

Across all studies, when provided, there was a general mention of the theoretical framework for the interventions. While all studies (11/11, 100%) named the parenting skills used, only a few (4/11, 36%) provided comprehensive and specific information about specific parenting strategies (eg, examples of the language used or a description of the applications of skills to in vivo situations) [51,52,57,58]. These studies discuss examples of the applications of individual parenting skills to daily life situations [51,58] or specific language used to deliver the skill [52,57].

#### Theoretical Frameworks Promoting Behavior Change Within a Mobile Platform

Only 2 (18%) of the 11 studies described the use of behavior change theories in the design of the mobile intervention for parents [51,53], which included social cognitive theory [53] and self-determination theory [51]. Details about these frameworks were found by reviewing preliminary, formative research [31,51,53]. No other study provided information about behavior change theories having guided the mobile intervention design. Of note, Schaeffer et al [58] have a manuscript in preparation that aims to describe the development of the mobile app. As such, this manuscript under preparation may allude to the behavior change theories that underlie the mobile app development. Nonetheless, the lack of behavior change theory implementation in mobile interventions is consistent with findings from past reviews [33,34,63], suggesting that designing mobile phone–based interventions without a theoretical foundation for behavior change within design is a common practice across different niches in the mobile intervention literature.

However, an evaluation of the studies using the taxonomy of behavior change techniques [46] revealed that these techniques were frequently used. The number of behavior change techniques included in the interventions ranged from 2 [49] to 9 [51]. The most used behavior change techniques within the apps included providing instruction (9/11, 82%), prompting practice (9/11, 82%), and prompting self-monitoring of a behavior (8/11, 73%). Taken together, the studies seem to have implemented some behavior change techniques widely, but the techniques were not guided by a stated behavior change theory in most of the studies (9/11, 82%; Table 2).

**Table 2:** Characteristics and features of reviewed studies.

Study, year	Sample, n; design	Delivery medium	Stand-alone treatment or an enhancement to treatment	Mobile device used	Mental health condition targeted	Type of intervention used, incorporated, or adapted	Underlying intervention theory	Behavior change theory applied in design	Behavior change techniques	Parent training skills used in intervention	Youth outcomes	Parent outcomes
Breitenstein et al [53], 2016	79; RCT^a^	MA^b^	Stand-alone treatment	Tablet	Behavior problems	Chicago Parent Program	CM^c^, SLT^d^, and SCT^e^	SCT (theory was specified in a referenced article or preliminary work that was referenced)	CR^f^, GE^g^, M or D^h^, OSC^i^, PF^j^, PI^k^, PP^l^, and SM^m^	Parent-child relationship; clear expectations and rules; rewards and incentives; setting behavior goals; and effective requests	No significant change in child behavior problems	Improvement in parental warmth (Cohen *d*=0.31); improvement in parental self-efficacy (Cohen *d*=0.13), and improvement in parental follow-through on skills (Cohen *d*=0.18)
Feil et al [54], 2018	42; RCT	MA	Stand-alone treatment	Smartphone	Conduct and antisocial behaviors	Behavioral parent training skills	—^n^	—	FU^o^, GE, PP, RG^p^, SGS^q^, and SM	Clear expectations and rules; rewards and incentives; setting behavior goals	—	No significant change in parenting behaviors
Hemdi and Daley [55], 2016	62; RCT	MA (the app used was an existing messenger app, not a newly developed one)	Enhancement to treatment	Smartphone	Autism spectrum disorder	Psychoeducation intervention	DABCX^r^ and TMS^s^	—	PIC^t^, PIN^u^, and SM	Parent-child relationship	Improvement in hyperactivity (Cohen *d*=–1.58)	Reduction in parenal stress (Cohen *d*=–0.98); reduction in parental depression (Cohen *d*=–2.05)
Jones et al [51], 2014	15; RCT	SPE^v^	Enhancement to treatment	Smartphone	Disruptive behavior disorders	Helping the Noncompliant Child	SLT and CM	SDT^w^ (theory was specified in a referenced article or preliminary work that was referenced)	BI^x^, CR, GE, M or D, OSC, PF, PI, PIN, PP, and SM	Parent-child relationship; clear expectations and rules; rewards and incentives; effective requests; praise; planned ignoring; modeling	Improvements in intensity of disruptive behaviors (Cohen *d*=0.99); improvements in presence of disruptive behaviors (Cohen *d*=0.54)	Improvement in parental engagement and generalization of parenting skills (Cohen *d*=0.88); increased participation in midweek check-ins (Cohen *d*=2.59); increased completion of home practice (Cohen *d*=0.63)
Lefever et al [56], 2017	371; RCT	SMS text messaging	Enhancement to treatment	Mobile phone	Behavior problems	Parent Child Interaction module of SafeCare	SLT and EST^y^	—	GE, PF, PI, and PP	Parent-child relationship; clear expectations and rules; rewards and incentives; praise; modeling	Improvement in cooperative behavior (Cohen *d*=0.38)	Increase in observation of parenting skills use (Cohen *d*=0.68); improvement in responsive parenting skills (Cohen *d*=0.35); growth of use of parenting skills (Cohen *d*=0.28)
Mason et al [57], 2021	52; RCT	SMS text messaging	Stand-alone treatment	Mobile phone	Substance use	Behavioral parent training skills	SLT, SCT, and CM	—	IF^z^, PIC, RG, and PI	Parent-child relationship; effective requests; monitoring	Decrease in depressive symptoms (Cohen *d*=–0.63); decrease in anxiety symptoms (Cohen *d*=–0.57)	Improvement in parental relations (Cohen *d*=0.41); improvement in parenting skills (Cohen *d*=0.51)
May et al [50], 2021	184; pilot study	SMS text messaging	Stand-alone treatment	Mobile phone	Autism spectrum disorder	Behavioral parent training skills^aa^	—	—	PIN and GE	Parent-child relationship	—	Improvement in parent-child relationship
Pina et al [52], 2014	10; pilot study	MA; EDA wristband^ab^	Stand-alone treatment	Mobile phone; tablet	Attention-deficit/hyperactivity disorder	Parental behavioral therapy^aa^	SLT and TTC^ac^	—	GE, IF, IRM^ad^, PI, PP, and SM	Parent-child relationship; clear expectations and rules; setting behavior goals; effective requests; praise; planned ignoring; modeling	—	—
Schaeffer et al [58], 2022	72; RCT	MA	Stand-alone treatment	Smartphone	Conduct problems	Multisystemic therapy	EST	— (app did not explicitly state theory but suggested the presence of a theory without providing content indicating that the theory was used in this study)	PP, SM, IF, M or D, PI, PIN, RG, SGS, and CR	Parent-child relationship; clear expectations and rules; rewards and incentives; setting behavior goals; effective requests; monitoring; modeling	Decrease in substance use, delinquency, and status offenses (Cohen *d*=0.54-0.84)	Improvement in discipline consistency (Cohen *d*=0.44); improvement in rule clarity (Cohen *d*=0.32)
Sonne et al [59], 2016	11; pilot study	MA	Stand-alone treatment	Smartphone	Attention-deficit/hyperactivity disorder	The Incredible Years Program^aa^	SLT	— (app did not explicitly state theory but suggested the presence of a theory without providing content indicating that the theory was used in this study)	IF, PI, PP, SGS, and SM	Parent-child relationship; clear expectations and rules; rewards and incentives; setting behavior goals; monitoring	Reduction in inattention (Cohen *d*=0.73); improvement in conduct-related behaviors (Cohen *d*=1.02); improvement in youth sleep (Cohen *d*=0.67)	Improvement in parental frustration
Sullivan et al [60], 2019	45; pilot study	MA	Enhancement to treatment	Smartphone	Trauma	RPC^ae^ and TIPS^af^	AT^ag^, CBT^ah^, CDT^ai^, SLT, and RT^aj^	— (app did not explicitly state theory but suggested the presence of a theory without providing content indicating that the theory was used in this study)	CR, M or D, OSC, PI, PIN, PP, and SM	Parent-child relationship; clear expectations and rules; praise; planned ignoring	Increase in youth prosocial behavior (Cohen *d*=0.40)	Improvement in parental self-efficacy (Cohen *d*=0.41)

^a^RCT: randomized controlled trial.

^b^MA: mobile app.

^c^CM: coercion model.

^d^SLT: social learning theory.

^e^SCT: social cognitive theory.

^f^CR: providing contingent rewards.

^g^GE: providing general encouragement.

^h^M or D: behavior modeled or demonstrated by a professional.

^i^OSC: opportunities to view social change.

^j^PF: providing feedback.

^k^PI: providing instruction.

^l^PP: prompting practice.

^m^SM: self-monitoring of specific behavior.

^n^Not applicable (not reported or not able to draw from study information).

^o^FU: providing follow-up prompts.

^p^RG: prompting a review of current goals.

^q^SGS: specific goal setting.

^r^DABCX: Double ABCX Model of Stress.

^s^TMS: Transactional Model of Stress.

^t^PIC: providing information on consequences of behaviors.

^u^PIN: providing information.

^v^SPE: smartphone enhancements, including SMS text messaging, video calls, alarms, and skills videos.

^w^SDT: self-determination theory.

^x^BI: barrier identification.

^y^EST: ecological systems theory.

^z^IF: prompting intention formation.

^aa^Intervention design was based on user and professional feedback but drew on elements of the mentioned intervention.

^ab^EDA: electrodermal activity.

^ac^TTC: transtheoretical change theory.

^ad^IRM: prompting identification as a role model.

^ae^RPC: Resource Parent Curriculum (National Child Traumatic Stress Network).

^af^TIPS: trauma-informed parenting skills.

^ag^AT: attachment theory.

^ah^CBT: cognitive behavioral theory.

^ai^CDT: child development theory.

^aj^RT: resilience theory.

### Design Elements

#### Features

Assessment of all the studies suggested the presence of 5 features: tailoring intervention content, push notifications, tracking of behaviors, modeling skills through video demonstration, and reward systems. The reviewed studies varied in their implementation of these features. First, many of the studies (7/11, 64%) included options for the end user to tailor mobile app intervention content or features. Options to tailor intervention content included defining individualized behavioral goals such as completing household chores, following a bedtime routine, returning home by curfew, and completing homework (4/7, 57%) [53,54,58,59]; creating a schedule for when to use the parenting skills provided in the app (eg, choosing when to engage in particular modules, creating a routine for parents, and allowing ongoing access to psychoeducation; 3/7, 43%) [53,59,60]; delivering just-in-time interventions according to individual stress level (2/7, 29%) [52,58]; selecting rewards or contingencies that they think their child would value (4/7, 57%) [53,54,58,59]; and receiving psychoeducation tailored to individual circumstances (eg, how to intervene when the child is in a risky situation and using time-outs with children who have experienced trauma; 4/7, 57%) [51,53,58,60]. Several studies also offered opportunities to tailor features of the app (6/11, 54%). Options to tailor app features included choosing icons, avatars, and profile photos that embody the user (2/11, 18%%) [54,60]; filming oneself practicing skills with the youth (1/11, 9%) [51]; and integration of the user’s name in the delivery medium (4/11, 36%) [54,57-59]. Notably, only a few of the studies (2/11, 18%) provided comprehensive visual examples or a description of the treatment content and mobile platform. As a result, other design features may be embedded in the apps but have not been identified in this review.

Second, most of the mobile interventions (8/11, 73%) included push notifications and SMS text messages to prompt practice of strategies or provide reinforcement and encouragement [50-52,55-59].

Third, some of the studies (3/11, 27%) included a mechanism for tracking youth behaviors [54,58,59], such as completing steps in a routine, monitoring the youth’s location, and assessing the completion of positive behaviors. Behaviors were tracked either by parents [60] or by both parents and children [54,58,59], and they were logged by adding events to a log sheet [54,58] or by moving through a checklist in situ [59]. Behavioral tracking (ie, assessing and following the behaviors of the youth concerned) was implemented through the mobile intervention in each of these studies.

Fourth, some of the studies (4/11, 36%) included videos modeling parent-child interactions and other parenting skills [51,53,58,60].

Finally, nearly half of the studies (5/11, 45%) featured a reward system for either the parent or the child [53,54,58-60] that was implemented through the mobile intervention. For children, rewards included points [54,58] and stickers [59], while parents earned completion badges and certificates [53] or accessories for an avatar family [60].

#### Content

Broadly, the mobile intervention content related to behavioral parent training skills included strengthening the parent-child relationship (10/11, 91%; the exception was the study by Feil et al [54]), setting clear expectations and rules (8/11, 73%; the exceptions were the studies by May et al [50], Mason et al [57], and Hemdi and Daley [55]), the establishment of rewards and incentives (6/11, 55%) [51,53,54,56,58,59], setting behavioral goals for the youth (5/11, 45%) [52-54,58,59], the use of effective communication and requests (5/11, 45%) [51-53,57,58], praising desired behaviors (4/11, 36%) [51,52,56,60], modeling effective behaviors (4/11, 36%) [51,52,56,58], planned and active ignoring (4/11, 36%) [51,52,56,60], and the implementation of monitoring and supervision (3/11, 27%) [57-59]. Although an indication of behavioral parent training skills can be gleaned from the description of the intervention, it is challenging to determine the exact number of these skills. This difficulty stems from a lack of detailed information in the articles regarding the specific skills provided in the mobile intervention. Of note, some of the studies (2/11, 18%) [51,56] involved enhancements to in-person treatment delivery, suggesting that additional skills were likely provided and discussed through the technology, although they were not explicitly mentioned in the manuscripts.

Ideally, a review of app content includes an assessment across 4 domains: evidence-based content, quality of information provision, completeness and conciseness, and clarity about the program’s purpose [43]. To fully implement this evaluation, studies must provide comprehensive information, including examples of content across the intervention (eg, specific messages designed for the end user, video dialogue, and prompts used to encourage practice). Unfortunately, most of the studies (10/11, 90%) included in this review did not include enough information for a thorough review of app content across these 4 dimensions. In fact, only 1 (9%) of the 11 studies [52] allowed for a partial evaluation of content according to the Enlight domains.

For 8 (73%) of the 11 studies, specific, direct content was not described [50,51,53-56,58,60]. Consequently, the content could not be evaluated against the Enlight domains.

Of the 11 studies, 3 (27%) provided specific examples of content [52,57,59]. However, the information was not comprehensive in that it consisted of brief sample treatment statements and lacked psychoeducation related to the skills being implemented. For 2 (67%) [57,59] of these 3 studies, none of the Enlight domains could be evaluated. With regard to the third study [52], some Enlight domains were assessed (ie, clear and concise goals, quality information necessary to obtain these goals, and clarity regarding the purpose and target population of the program). The evaluation showed that the content fulfilled these domains at *good* levels.

#### Visual Design

Ideally, a review of visual design includes the evaluation of aesthetics, layout, and size [43]. As with content evaluation, studies must provide a comprehensive collection of visualization of the mobile app. Because this assessment examines font consistency, the harmony of colors used throughout, and the size of the layout on the mobile device [43], a comprehensive set of visualizations would include examples of various pages in the app design and include examples of color, font, images, and treatment content. Most of the studies (6/11, 54%) included in this review provided few visualization examples, which created difficulty in conducting a full assessment of the visual design used throughout the app.

Of the 11 studies, 4 (36%) were not evaluated for visual design because either the interventions were SMS text message based [50,56,57] or no visual information was provided in the article [55]. Among the remaining 7 studies, 6 (86%) included examples of the mobile intervention with snapshots of select screens, rather than a visual design sample of the app in its entirety [52-54,58-60]. Of note, 1 (17%) of these 6 studies included visualizations of the mobile app through a website, rather than within the published study article [58]. Because of the scarcity of visual examples among the studies included in this review, evaluation based on the Enlight criteria was completed for the components of the intervention the researchers selected to present, rather than the app in totality. The results showed that the aesthetics classifications included *not attractive* (1/6, 17%), *fair* (1/6, 17%), *attractive* (2/6, 33%), and *very attractive* (2/6, 33%). The layout classifications ranged from *fair* (1/6, 17%) and *good* (4/6, 67%) to *very good* (1/6, 17%). The size qualities ranged from *fair* (1/6, 17%) and *good* (2/6, 33%) to *very good* (3/6, 50%).

Most of the studies (4/11, 36%) implemented muted colors on certain screens or activities for parent-directed content [52,53,59,60]. For parent- and child-directed or child-only–directed content, colors were brighter than those seen on parent-directed content screens [54,59]. Using brighter colors with children aligns with the robust literature on the preference of younger children for brighter, more saturated colors over more muted colors [64-66]. The depiction of families was an overwhelmingly common visual design element; for example, the studies included photos of families on home pages who resembled the families using the app [53], actual photos of the families themselves [54], or the integration of the names of the children [58].

### Treatment Outcomes

#### Youth Outcomes

Of the 11 studies, 8 (73%) provided youth outcomes. Of these 8 studies, 7 (88%) indicated substantial improvement in the youth. Of these 7 studies, 6 (86%) were randomized controlled trials and indicated that the youth showed greater improvement in the technology-enhanced group when compared to the control group [51,53,55-58]. Overall, the youth in these randomized trials exhibited a decrease in behavioral and mood-related problems [51,55,56,58]; for example, Lefever et al [56] reported that youth with parents in the intervention condition demonstrated a significant improvement in cooperative behavior with a small to medium effect size (Cohen *d*=0.38); Hemdi and Daley [55] reported significant improvement in hyperactivity for youth in the intervention condition with a large effect size (Cohen *d*=−1.54); Jones et al [51] reported significant improvements in the intensity (Cohen *d*=0.99) and presence of disruptive behaviors (Cohen *d*=0.54) with medium to large effect sizes for youth in the intervention condition; Mason et al [57] reported significant small to medium effect sizes, demonstrating a decrease in depressive (Cohen *d*=−0.63) and anxiety (Cohen *d*=−0.57) symptoms; and Schaeffer et al [58] reported significant decreases in substance use, delinquency, and status offenses for youth in the intervention condition with medium to large effect sizes (ranging from Cohen *d*=0.54 to Cohen *d*=0.84).

Of the 8 studies, 2 (25%) were pilot studies [59,60]. Although these studies did not use a randomized group as a comparison, both reported similar improvements in youth behavior problems; for example, Sonne et al [59] reported a significant reduction in inattention at a medium effect size (Cohen *d*=0.73), improvement in conduct-related behaviors at a large effect size (Cohen *d*=1.02), and improvement in youth sleep at a medium effect size (Cohen *d*=0.67). Sullivan et al [60] reported an increase in youth prosocial behavior at a small effect size (Cohen *d*=0.40).

#### Parent Outcomes

Most of the studies (10/11, 91%) reported parent outcomes. Many of the studies (9/11, 81%) reported parental improvements when using mobile technology. Among the 7 studies using a randomized controlled trial design, all (7/7, 100%) reported parental improvements in the technology intervention groups compared to their respective control groups. Specifically, Breitenstein et al [53] reported an improvement with small to medium effect sizes in parental warmth (Cohen *d*=0.31), parental self-efficacy (Cohen *d*=0.13), and parental follow-through on skills (Cohen *d*=0.18). Hemdi and Daley [55] reported large effect sizes for reduction in parental stress (Cohen *d*=−0.98) and parental depression (Cohen *d*=−2.05) among parents in the mobile intervention group. Lefever et al [56] reported a medium effect size in the observation of parenting skills use (Cohen *d*=0.68), a small to medium effect size in the improvement in responsive parenting skills (Cohen *d*=0.35), and a small effect size in the growth of use of parenting skills (Cohen *d*=0.28). Jones et al [51] reported a greater improvement in parental engagement and the generalization of parenting skills for the parents using a mobile intervention with weekly attendance (Cohen *d*=0.88), participating in midweek check-ins (Cohen *d*=2.59), and the completion of home practice (Cohen *d*=0.63), reflecting medium to large effect sizes. Schaeffer et al [58] reported small to medium effects in the improvement of discipline consistency (Cohen *d*=0.44) and rule clarity and structure (Cohen *d*=0.32). Mason et al [57] reported small to medium effect sizes in the improvements of parent relations (Cohen *d*=0.41) and parenting skills (Cohen *d*=0.51), reflecting medium effect sizes. Finally, and of note, while Feil et al [54] reported a small to medium effect size in the reduction of negative parenting behaviors, the researchers note that this finding is insignificant and did not report this coefficient. Therefore, this reported finding is not included in Table 2.

Of the 10 studies, 3 (30%) were pilot studies that incorporated parent outcomes. Although these studies did not use a randomized group as a comparison, they described a small effect size in the improvement of parental self-efficacy (Cohen *d*=0.41) [60], significant improvements in parental frustration [60], and improvements in parent-child relationship [50] among the participants engaging in the mobile intervention.

## Discussion

### Overview

This study systematically reviewed noncommercial mHealth apps that provide behavioral parent training or components of behavioral parent training for parents of children with behavior problems. This study had the specific goals of summarizing (1) general characteristics, (2) theoretical frameworks and empirical evidence, and (3) parenting strategies. The broad aim for this review was to use the results to inform the development of an app for parents of teens who are involved in substance use behaviors.

### Use of Theory

This review found that all studies (11/11, 100%) included in this review used parent training interventions that are theoretically grounded. However, there was a paucity of clear information outlining the theoretical framework for the components designed within the mobile apps. While many of the apps were based on face-to-face parent management training interventions that have well-established theoretical frameworks (eg, Helping the Noncompliant Child, Parent-Child Interaction Therapy, and Parent Management Training), the studies often only referenced the interventions [56,57,59,60] or reported specific parenting skills without specifying the originating intervention [50,54,55]. Determining relevant theoretical frameworks required deductive reasoning based on a knowledge of the named intervention or parenting skill. Using this expertise, we found that many of the apps (6/11, 55%) used social learning theory [67], while only a few (4/11, 36%) [51-53,59] indicated the use of the coercion model [62], either by mentioning this model by name [51,53] or by describing the benefits of the parenting skills that were selected for inclusion in the apps [52,59].

Given that parent training interventions can draw from different theoretical frameworks beyond social learning theory and the coercion model [68] and that the full in-person treatment programs were not transferred to the mobile apps in the reviewed studies, implicit communication of the theoretical framework through only naming the originating intervention or specific skills is not sufficient. It is important to clearly state the relevant theoretical framework for the content transferred to mobile devices because the inclusion of a particular intervention does not guarantee that its principles are embodied in the mobile platform. A deeper analysis of whether specific interventions are consistent with the theory in terms of mobile app features could not be performed due to a lack of information.

### Behavior Change

Although behavior change theory is a vital component of mobile interventions [33,34,62,69], the results of this review reveal that most of the studies (9/11, 81%) did not explicitly refer to behavior change theory. The absence of behavior change theory as a framework for app design in many of the reviewed studies (9/11, 81%) may be due to at least 3 reasons. First, many of the studies (8/11, 72%) failed to provide sufficient information about the content and development of the mobile intervention, making it challenging to understand the detailed study characteristics. Second, the studies may have relied on behavior change theories for the originating curriculum due to the well-established programs on which the interventions are based. However, this overlooks the challenges of transferring the interventions to mobile devices. Many parent management training curricula incorporate behavior change theories that consider factors such as personal motivation, social support, and perceived barriers and benefits of behavior change. While similar theories may be used in mHealth interventions, there are additional considerations, such as the need to focus more on technology-specific behavior change theories; for example, the technology acceptance model [70] focuses on how individuals perceive and adopt novel technologies, and it may be a suitable theory to embed in mHealth development. The lack of prior designs centered on the individual’s perspective remains a gap. Third, there may have been a general oversight in including a coherent behavior change theory in the intervention’s design. Given the impact of behavior change theory on the effectiveness of interventions [33,34,69,71], its inclusion is crucial for the development of effective parent-targeted mobile interventions.

Although behavior change theories were not commonly cited in the reviewed studies, behavior change techniques were used. The most used techniques were providing instruction, prompting practice, and self-monitoring [46]. The findings of this study are consistent with prior reviews showing that self-monitoring was the most frequently used behavior change technique in mobile interventions across different populations [33,35]. In this review, self-monitoring described tracking both the implementation of parental skills and behaviors and the presence of desired behaviors in children. The limitations of the studies in terms of behavior change theory and techniques will be discussed further while summarizing the design elements (refer to the next subsection).

### Mobile Intervention Design

In this review, we evaluated the design of mobile interventions used in the included studies based on their content, visual design, and features. While all studies incorporated particular parenting skills (11/11, 100%), comprehensive information regarding the implementation of specific skills was not included in many of the studies (4/11, 36%). When information was provided, there was limited detail on the selection and integration of the skills into the mobile platform. This lack of information made it challenging to critically assess these interventions and consider them in their entirety for use with different populations, such as parents of adolescents recovering from substance use.

The visual design and features of the mobile interventions in the included studies were designed to be personalized. Each mobile intervention had its own way of promoting personalization through visual design and features; for example, users could customize the visual design by selecting icons, fonts, and colors that were personally appealing [53,54,59]. This practice of personalizing the layout and design has also been noted in previous literature reviews [72], suggesting consistent focus across populations and areas of study. The features of these mobile interventions also facilitated individuality, including avatar families [60], tailored messages [56], and the option to choose specific skills to practice based on individual needs [53].

In addition, the use of badges, rewards, logs, and tokens as a feature in the reviewed mobile interventions was a common pattern This feature was also noted in previous reviews as prevalent [72]. The use of rewards aimed at positively reinforcing desired behaviors in both parents and adolescents and encouraged individualization. Positive reinforcement, in which a stimulus increases the frequency of a particular behavior, is a well-established behavior change technique [73]. When implemented within a structured framework, positive reinforcement can be effective in promoting desired behaviors [73].

### Treatment Outcomes

The preliminary findings of the reviewed studies indicate potential for positive parent and child outcomes after the use of a behavioral parent training app [50,51,55-60], but further research is necessary to support these findings. Most of the studies that reported on youth and parent outcomes (8/11, 72%) used interventions that were grounded in well-established theoretical frameworks [51,53,55-60], suggesting that theory-driven interventions may play a critical role in outcomes after behavioral parent training delivered through mobile devices.

To optimize the effectiveness of behavioral parenting apps, future studies should incorporate behavior change theories in the design and development process. The limited information available in previous studies on the content and development of parent-targeted interventions within mobile platforms makes it challenging to identify the behavior change theory applied, if any (the exceptions were the studies by Jones et al [31], Jones et al [74], May et al [75], Breitenstein et al [53], Schaeffer et al [58], Jones et al [51], and May et al [50]). Thus, further empirical research is necessary to determine the influence of behavior change theory on the outcomes of behavioral parent training delivered through mHealth apps.

### Implications for mHealth Parenting Apps to Address Teen Substance Use

The development of behavioral parent training apps for parents of children with mental health difficulties is still in the preliminary stages, with available apps developed primarily for parents of younger children. However, there is a need for apps for parents of adolescents with conduct problems, including substance use. These apps are crucial because effective parenting strategies are related to decreased levels of substance use [2,3], and engagement in mobile platforms may be helpful for parents with difficulty accessing treatment in the community. Studies have revealed that it is challenging for parents to both access and engage in evidence-based treatments with behavioral parenting strategies in community settings [76,77].

While mHealth platforms provide accessibility, customization is key to fostering engagement. Thankfully, most of the apps (7/11, 63%) in this review of mHealth apps for delivering behavioral parent training included features such as customization and personalization, which are considered good practice [78,79]. However, the reviewed apps lacked integration within the app and between the app and smartphones. This limitation is due to the predominant focus on parents of younger children. However, as parents spend less time with their children, and key parenting strategies broaden to include monitoring and supervision during adolescence [80], the integration of features is likely to become increasingly important for behavioral parent training apps. To address this gap, it is recommended that a more comprehensive integration of app design and mobile phone features occur for apps targeting parents of adolescents; for example, location-based reminders could be used to track a teen’s location and send reminders or prompts relevant to their current location to the parent because parents often either rely on youth reports of their location or do not check in on potential location changes. Specifically, if the teen is at a location where they are likely to use substances, the app could send a reminder for the parent to have a preplanned conversation with their teen or to check up on their whereabouts. In addition, geofencing could be used to set up web-based boundaries around specific locations, sending an alert or reminder when the teen enters an *off-limits* location and offering suggestions for which parenting strategies to use to address the infraction. Alternatively, an alert with scripted language that is consistent with effective praise [21] could be sent to the parent if the teen’s movements suggest that an off-limits area was avoided so that the parent can engage in providing praise in the moment because offering immediate praise and feedback is key for changing behavior [73]. Notably, Schaeffer et al [58] used similar location-based strategies to encourage the monitoring and supervision of adolescents.

In addition, an app could be designed to use GPS data to generate reports that summarize the adolescent’s whereabouts over time. These reports could serve as personalized feedback with recommendations to the parents. This sort of analysis could help parents discern patterns and areas that require the use of certain parenting strategies. Moreover, push notifications could be integrated to remind parents to use a specific parenting strategy at a designated time or in response to a trigger that was defined by the personalized feedback report. Rich integration of features may bring social learning theory and the coercion model to the forefront, resulting in the potential integration of behavior change theories and techniques. If so, this integration could address limitations in most current noncommercial apps.

### Limitations and Strengths

It is important to consider the results of this review within the context of a few limitations. First, this review only looked at a specific area of mHealth research: mobile phone–based interventions that were designed to provide behavioral parenting practices to parents of children with mental health difficulties. Studies on mobile interventions targeting parenting for parents of children with medical issues were not included in this review. Given the overlap in issues related to ineffective parenting between parents of children with behavior problems and parents of children with chronic medical conditions such as asthma and obesity [81], future studies may benefit from reviewing parenting apps designed for both groups for a broader view. Second, this review was based on a new field of research. As such, there was a paucity of studies available for review. As a result, we included studies involving children of different ages, spanning different developmental windows and parenting needs. Notably, 2 (18%) of the 11 studies provided insight into current behavioral parenting apps for parents of teens [57,58]. Third and last, because of the small pool of studies, synthesis of data to obtain overall effect sizes was not possible. Therefore, as the field grows, the effectiveness of parent-targeted mobile interventions for parents of youth with mental health issues should be empirically assessed using statistical analyses to develop a meta-analysis. The garnering of data will provide more robust evidence of the effectiveness of these interventions in a population of youth with mental health difficulties.

Despite these limitations, this review has some strengths that make it valuable for understanding current noncommercial parenting apps for informing the development of similar apps for related problems in childhood and adolescence. By focusing on behavioral parenting apps for parents of children and adolescents with mental health difficulties, this review provides targeted and relevant information for developers who are interested in designing an app using parenting practices that are well established for other behavior problems occurring in youth, including substance use [2-4,7,82]. Finally, the results of this review provide clear information about current practices and patterns so that future research can more closely align the development of apps with design features that may increase treatment engagement [72,83] and, hopefully, buttress outcomes.

## Appendix

Multimedia Appendix 1 PRISMA (Preferred Reporting Items for Systematic Reviews and Meta-Analyses) checklist.

## Abbreviations

mHealth: mobile health
PRISMA: Preferred Reporting Items for Systematic Reviews and Meta-Analyses
